# Assessing the Level of Understanding (Knowledge) and Awareness of Diagnostic Imaging Students in Ghana on Artificial Intelligence and Its Applications in Medical Imaging

**DOI:** 10.1155/2023/4704342

**Published:** 2023-06-15

**Authors:** James William Ampofo, Christian Ven Emery, Ishmael Nii Ofori

**Affiliations:** Department of Imaging Technology and Sonography, School of Allied Health Sciences, College of Health and Allied Health Sciences, University Cape Coast, Cape Coast, Ghana

## Abstract

**Introduction:**

Recent advancements in technology have propelled the applications of artificial intelligence (AI) in various sectors, including healthcare. Medical imaging has benefited from AI by reducing radiation risks through algorithms used in examinations, referral protocols, and scan justification. This research work assessed the level of knowledge and awareness of 225 second- to fourth-year medical imaging students from public universities in Ghana about AI and its prospects in medical imaging.

**Methods:**

This was a cross-sectional quantitative study design that used a closed-ended questionnaire with dichotomous questions, designed on Google Forms, and distributed to students through their various class WhatsApp platforms. Responses were entered into an Excel spreadsheet and analyzed with the Statistical Package for the Social Sciences (SPSS) software version 25.0 and Microsoft Excel 2016 version.

**Results:**

The response rate was 80.44% (181/225), out of which 97 (53.6%) were male, 82 (45.3%) were female, and 2 (1.1%) preferred not to disclose their gender. Among these, 133 (73.5%) knew that AI had been incorporated into current imaging modalities, and 143 (79.0%) were aware of AI's emergence in medical imaging. However, only 97 (53.6%) were aware of the gradual emergence of AI in the radiography industry in Ghana. Furthermore, 160 people (88.4%) expressed an interest in learning more about AI and its applications in medical imaging. Less than one-third (32%) knew about the general basic application of AI in patient positioning and protocol selection. And nearly two-thirds (65%) either felt threatened or unsure about their job security due to the incorporation of AI technology in medical imaging equipment. Less than half (38% and 43%) of the participants acknowledged that current clinical internships helped them appreciate the role of AI in medical imaging or increase their level of knowledge in AI, respectively. *Discussion*. Generally, the findings indicate that medical imaging students have fair knowledge about AI and its prospects in medical imaging but lack in-depth knowledge. However, they lacked the requisite awareness of AI's emergence in radiography practice in Ghana. They also showed a lack of knowledge of some general basic applications of AI in modern imaging equipment. Additionally, they showed some level of misconception about the role AI plays in the job of the radiographer.

**Conclusion:**

Decision-makers should implement educational policies that integrate AI education into the current medical imaging curriculum to prepare students for the future. Students should also be practically exposed to the various incorporations of AI technology in current medical imaging equipment.

## 1. Introduction

The term artificial intelligence (AI) was coined by John McCarthy in 1956, for which he defined it as “the science and engineering of making intelligent machines”.

It is a broad term that describes the theory and development of computer systems that can perform activities that would ordinarily require human intellect, such as visual perception, voice recognition, decision-making, and language translation [1]. The applications of AI can be divided into two categories, namely: the endeavor to replicate human mental capacities and the invention of tools to carry out tasks that currently require human intervention [2]. The ability of AI to reason and act to attain a certain objective is its greatest strength [3]. AI is a rapidly evolving technology with a wide range of uses in society. It is gaining traction in a variety of industries, including healthcare, telecommunications, transportation, education, and law, among others. It enables them to make better decisions and increase productivity [4]. There are concerns about whether people are aware of the complexities of these applications [5].

AI has infiltrated practically every aspect of our daily lives without our knowledge, beginning with virtual assistants like Amazon's Alexa and Apple's Siri, face recognition, healthcare systems, self-driving cars, robotics, and so on [3]. The applications of AI in healthcare continue to pique people's interest [6]. The application of artificial intelligence in healthcare holds great promise for expanding medical knowledge and providing optimal yet cost-effective healthcare solutions [7]. In the clinical domain, expected outcomes include identifying individuals at high risk for a disease, improving diagnosis and matching of effective personalized treatment, and monitoring therapy response outside of the hospital [8, 9].

The use of artificial intelligence in medical imaging, specifically in image processing and interpretation, is one of the most promising areas of health innovation [10]. By increasing image acquisition, image assessment, and workflow speed, artificial intelligence (AI) is projected to change radiology practice [11, 12]. The benefits of AI in medical imaging are based on radiation risk, and AI algorithms can also be used in referral protocols and scan justification to help limit radiation exposure by eliminating unnecessary screening [13].

In medical imaging practice, AI has shown impressive precision and sensitivity in the identification and characterization of abnormalities, leading to improved service delivery and patient care quality [14]. However, as sensitivity improves, an important disadvantage emerges, namely the detection of subtle changes of uncertain significance [15]. An analysis of screening mammograms, for example, revealed that while artificial neural networks are no more accurate than radiologists in detecting cancer, they have consistently higher sensitivity for pathological findings, particularly subtle lesions [16].

Utilization of AI tools could ultimately lead to a reduction in radiation exposure while maintaining the high quality of medical images, although risks such as image distortion must be assessed [17]. The implementation of artificial intelligence for medical imaging by resource-poor health institutions is hampered by a lack of radiology resources. Local equipment, people expertise, infrastructure, data-rights regimes, and public legislation all constrain them [18].

## 2. Objectives

1. To determine the level of knowledge and understanding of diagnostic imaging students in Ghana about AI in the field of medical imaging
2. To investigate the impact of training and educational program on the level of knowledge and awareness of AI in medical imaging among diagnostic imaging students in Ghana
3. To provide recommendations for the integration of AI in the curricula of diagnostic imaging programs in Ghana
4. To assess the willingness of diagnostic imaging students to learn more about AI.

## 3. Materials and Methods

### 3.1. Study Design

A cross-sectional quantitative survey research was deployed for this study. The design provided the current level of awareness among a large number of imaging students and allowed the findings to be presented in the form of percentages and ratios. The data was collected in the course of the survey using a self-generated close ended questionnaire. The survey replaced presumptions with actual data from participants and examined the data from the participants. The study received ethical clearance from the Institutional Review Board (IRB) of the University of Cape Coast.

### 3.2. Study Area

The study was conducted among radiography students from the University of Cape Coast (UCC), the University of Ghana (UG), Kwame Nkrumah University of Science and Technology (KNUST), and the University of Health and Allied Sciences (UHAS) all of Ghana.

### 3.3. Study Population

The study was centered on second- to fourth-year radiography students enrolled in public universities in Ghana. They included both males and females. These categories of students were used because it was assumed that they already had a good idea about medical imaging. First-year students were excluded from the study population to get rid of possible bias since they were yet to take major Medical Imaging courses in the program and so did not have much knowledge about medical imaging and even AI in medical imaging and related AI applications. Guided by the reality that the whole population could not be used in the study, a confidence level of 95% was adopted.

### 3.4. Sampling and Sampling Technique

A stratified sampling technique was used for this study. The number of participants selected from each participating university was proportional to the percentage constituted by students in year 2 to year 4 in that university, to the total imaging student population in Year 2 to Year 4 in all the participating universities.

A total of 225 radiography students were done with the formula, *n* = (*N*/[1 + *N*(*e*^2^)]), from [19] where **n** = sample size **N** = population size **e** = level of precision chosen 0.05.225 diagnostic imaging students were used as sample size. A total number of 516 students were used as the sample frame for the sample size calculation, as shown in Table 1.

(1) $$\begin{array}{c} n=\frac{N}{\left[1+N\left(e^{2}\right)\right]}\text{,}\\ where\;N=516\text{,}\\ n=\frac{516}{\left[1+516\left(0.05^{2}\right)\right]}\text{,}\\ Sample\;size\;\left(n\right)=225.3\text{.} \end{array}$$

### 3.5. Sampling Procedure

The sample frame and sample sizes from the selected universities are presented in the Table 2.

### 3.6. The Stratified Sampling Method Was Carried Out in the following Steps

The population was initially categorized into strata based on universities, aiming to ensure representation from different institutions. Proportional allocation was employed to determine the number of students in each stratum, taking into account the overall population distribution. Specifically, UCC represented 27.91% of the population, KNUST represented 30.81%, UG represented 23.64%, and UHAS represented 17.64%. To determine the sample size for each stratum, the respective percentage was multiplied by the overall sample size of 225. Consequently, the sample sizes for UCC, KNUST, UG, and UHAS were calculated as 63, 69, 53, and 40, respectively, as shown in Table 2. To gather data, questionnaires were distributed to potential participants in each university. The process was closely monitored to ensure that the number of responses received aligned with the desired number of participants allocated to each university.

### 3.7. Research Instrument

The data collection tool used for the study was a self-generated closed-ended questionnaire with dichotomous questions. The questionnaire helped to obtain quantitative data for statistical analysis of the survey findings. The questionnaire was self-generated based on the research questions and after and after a careful review of the questionnaire used in [20] which relates to the topic of study.

To measure the validity and reliability of the questionnaire, it was pretested through a pilot study among 30 randomly selected nonmedical imaging students to check for language and the acceptability and feasibility of the study approach and also consistency of the results. The questionnaire was reviewed by subject matter experts at the Institutional Review Board at the University of Cape Coast before ethical clearance was given.

After a careful analysis of the results and comments from the pilot study, necessary corrections were then made, and the responses obtained were cleared using the “delete responses” command in Google Forms online before the actual data collection process began.

The questionnaire had two main sections: A and B. Section A contained the participant's information sheet, which explained the nature and purpose of the study and some terminologies in the study, the rights of the participants, and finally asked for their consent. Section B had five parts. Part 1 had questions that sought the biodata of participants, namely, age, sex, institution, and year. The participant's name was excluded for anonymity and confidentiality.

Part 2 contained questions on general knowledge on AI. Part 3 contained questions that assessed the participant's awareness on AI; Part 4 had questions that tested the participant's knowledge on AI in medical imaging. The final part, Part 5, evaluated the contribution of the clinical internship and curriculum to the participant's knowledge and awareness of AI. There were a total of 20 questions, and they were self-administered online using Google Forms. However, the questionnaire could not test for any relationship between the various variables.

Before the questionnaire was administered, approval was sought and received from the heads of the department of the participating universities.

### 3.8. Data Collection Procedure

Google Forms links were sent to potential participants in each participating university via WhatsApp. This was done by identifying participants through appropriate authorities and sending the questionnaire link to their WhatsApp contacts. The aim was to obtain a sample size of 225 which was a representation of the population of interest. However, it is important to acknowledge that, using WhatsApp as a mode of sharing the questionnaire link may have introduced some potential biases such as the possibility that some potential participants may have missed the message or chosen not to respond. So the results were interpreted with this limitation in mind. During data collection, confidentiality was ensured in several ways.

First, we used a secure online survey tool (Google Forms) that encrypted connections to protect data transmission. The questionnaire link was only made accessible to students who were eligible to participate. Second, the questionnaire was designed to allow participants to answer the questions anonymously, without providing any personally identifiable information such as name and e-mail. This helped protect the privacy of the students and made it more difficult to identify individual responses. Third, we provided a clear and concise explanation of the purpose of the study. Respondents were asked to provide informed consent before starting the questionnaire and were given the option to withdraw from the survey if they wished.

Finally, we ensured that all data collected through the questionnaire was stored securely and that only authorized personnel had access to the data. We took great care to comply with data protection regulations and ensure that the privacy of our participants was protected at all times.

Students were asked to read and the participant information page attached to the questionnaires before agreeing or declining to participate in the research. The questionnaire was designed to be completed on an average time of 5 minutes. Each participant could only answer once. Data collection was carried out between September and October 2022. To prevent third-party access, the data were encrypted and compressed. All data were saved on a Google Drive after processing for safety and security reasons.

### 3.9. Statistical Analysis

The collected data was analyzed using statistical software, specifically Statistical Package for the Social Sciences (SPSS) version 25.0 and Microsoft Excel version 2016. Descriptive and basic statistical analyses were conducted to examine the obtained results. For categorical variables such as age, sex, and educational level, percentages were calculated and presented. The findings were then presented through tables, frequencies, and graphs to provide a clear and comprehensive representation of the data. For this study, sophisticated statistical techniques were not necessary.

## 4. Results and Discussion

### 4.1. Results

The survey was conducted among 225 medical imaging students; 181 students completed the entire survey, generating a response rate of 80.44%. The study employed fundamental statistical methods to analyze the data collected, and the outcomes obtained are constrained in scope, primarily due to the nature of the data collected and the questionnaire employed. Moreover, basic statistical techniques were employed to establish a fundamental understanding of the knowledge and awareness levels of diagnostic imaging students concerning AI in medical imaging. This serves as a crucial groundwork for subsequent studies.

#### 4.1.1. Part 1: Respondent's Demographics

The survey collected data on various demographic and academic characteristics of the respondents. The findings revealed that 53.6% (97 out of 181) of the respondents were males, while 45.3% (82 out of 181) were females. Additionally, 1.1% (2 out of 181) chose not to disclose their gender. In terms of age, the majority of respondents, constituting more than 80% (145 out of 181), fell within the 20 to 23 years age range. Among them, the highest proportion, 26.5% (48 out of 181), was 22 years old, followed by 22.1% (40 out of 181) who were 21 years old.

When considering the institutions, the University of Cape Coast accounted for the highest number of responses, representing 39.2% (71 out of 181) of the total. On the other hand, the lowest number of responses came from the Kwame Nkrumah University of Science and Technology, comprising 17.7% (32 out of 181) of the respondents. Regarding academic year level, the majority of respondents were in Year 3, comprising 37.6% (68 out of 181) of the total. Following closely, year 2 accounted for 36.5% (66 out of 181) of the respondents. The lowest representation was found in year 4, with only 26.0% (47 out of 181) of the respondents belonging to that academic year.

#### 4.1.2. Part 2: General Knowledge about Artificial Intelligence

General knowledge about artificial intelligence is present in the Table 3.

#### 4.1.3. Part 3: Knowledge about Artificial Intelligence in Medical Imaging

**Figure 1:**
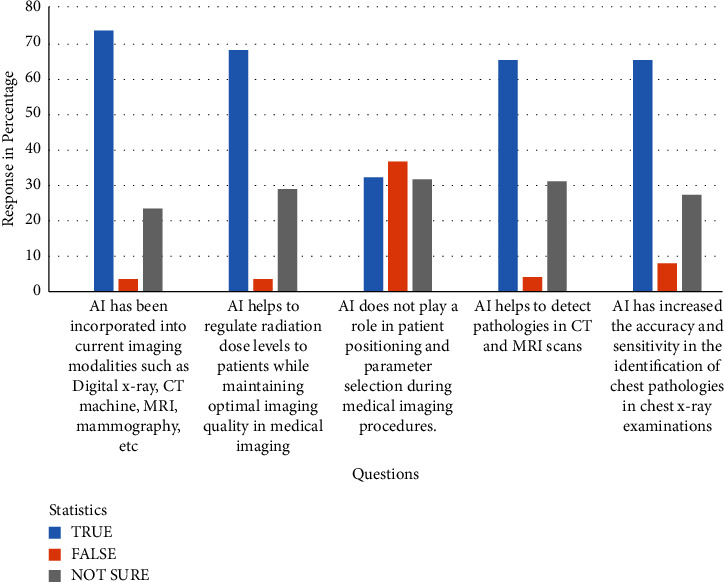
Distribution of the responses to knowledge about artificial intelligence in medical imaging.

Knowledge about artificial intelligence in medical imaging is present in the Figure 1.

#### 4.1.4. Part 4: Awareness of Artificial Intelligence in Medical Imaging

Awareness of artificial intelligence in medical imaging is present in the Table 4.

#### 4.1.5. Part 5: Clinical Practice and Curriculum

**Figure 2:**
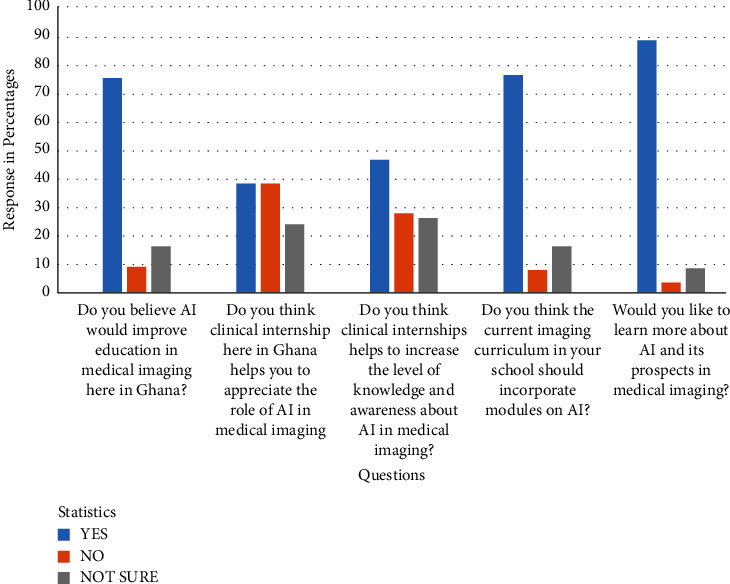
Distribution of responses to questions on impact of clinical practice and curriculum.

Clinical practice and curriculum is present in Figure 2.

## 5. Discussions

A national survey to assess the level of knowledge and awareness on artificial intelligence (AI) and its prospects in medical imaging was conducted between September and October 2022 among second- to final-year medical imaging students of four public universities in Ghana during the period September to October 2022. A total of 181 students, 53.6% males and 45.4% females with a mean age of (21.7) and SD of (1.81) with more than 80% (*n* = 145) between the ages of 20 and 23 years participated in the survey. Final-year students were the least represented (26%, *n* = 47); however, respondents from years 2 and 3 were almost equal (36.5%, *n* = 66 and 37.6%, *n* = 68, respectively). The available literature pertaining to students' knowledge regarding AI in medical imaging is limited in scope. This limitation led to the repetition of certain findings, thereby potentially influencing the discussion. Nonetheless, the scarcity of literature concerning students' knowledge about AI in medical imaging serves as a justification for undertaking this study.

### 5.1. General View on the Influence of Artificial Intelligence on Healthcare and Basic Knowledge

The field of medical imaging has seen significant advancements over the past few years, and with it, the integration of artificial intelligence (AI) has been widely accepted as a game-changer in medical practice [21]. The current study reveals significant findings regarding the perceptions of diagnostic imaging students in Ghana regarding the impact of artificial intelligence (AI) on the healthcare sector. As shown in Table 3, a majority of 81.2% (147 out of 181) expressed their agreement that AI is bringing about positive changes in healthcare. This result is consistent with a previous study conducted by researchers [6], which reported that 88% (432 out of 484) medical students from nineteen medical schools in the UK believed that AI would play a crucial role in the future of healthcare. Correspondingly, the research works of [22, 23] demonstrated that over 80% of the respondents in both studies believed that artificial intelligence would greatly benefit medicine and radiology as a whole.

Regarding concerns about the negative impact of AI on healthcare, the findings of this study indicate that 96.7% (175 out of 181) of the medical imaging students either believed or were uncertain about AI being a detrimental technology, as shown in Table 3. This suggests a relatively positive outlook among the participants towards AI in healthcare in Ghana.

In terms of basic knowledge about AI, as shown in Table 3, this study highlighted that 92.3% (167 out of 181) of the imaging students were aware of the full meaning of AI, indicating a solid foundation in understanding the concept. Additionally, 72.9% (132 out of 181) of the students expressed a general understanding of AI, which is coherent with the findings of studies [3, 24]. These studies reported that approximately 50% (238 out of 476) of medical students in Saudi Arabia and 62.3% (141 out of 226) of medical students in India claimed to possess knowledge about AI, respectively. However, a study conducted by [22] revealed that only 30.8% (81 out of 263) of undergraduate medical students had a basic understanding of artificial intelligence. This disparity may be attributed to the effectiveness of AI education, which has significantly improved in recent years.

As AI continues to evolve and improve, it is imperative that medical imaging students in Ghana are kept abreast of these developments and their potential prospects in medical imaging. Ghana is still in the early stages of AI implementation and, as such, there is limited native data available. However, with the increasing importance of AI in the field of medical imaging, it is crucial for Ghana's medical imaging students to stay informed and up-to-date on the latest advancements and their potential impact. By doing so, they will be better equipped to provide quality healthcare services to patients in the future.

### 5.2. Knowledge about Artificial Intelligence and Its Applications in Medical Imaging

The findings in Table 3 showed that almost all students (92.3%, 167/181) know the full meaning of AI, with a majority (72.9%, 132/181) of the students expressing that they have a general knowledge of AI, which is consistent with the findings of [25] where 78.9% of the respondents agreed to the question “I have a good understanding of what AI is” reflecting a high confidence level in their understanding of AI. In congruence, a study by [26] pointed out that the majority of the respondents, 75.0% (*n* = 201/268), expressed that they have some form of knowledge about AI. The positive results in these studies suggest that the increased emphasis on AI education in recent years may have contributed to the student's knowledge and understanding of AI.

On the contrary, only a few students (*n* = 31/181) in this study indicated that they do not have any general knowledge on artificial intelligence. The findings from the current study reveal a highly encouraging trend. A majority (71.8%, 130/181) of the students in Table 3 showed that AI is not a bad technology, indicating that AI will do more good than harm. However, students showed concern about job security in Table 3, where almost half (49.2%, 89/181) of them think that AI poses a threat to job vacancies and that it will one day replace humans which is in line the findings of [26] where 43.7% (*n* = 117/268) of the participants expressed they have concerns that radiographers and radiologists will lose their jobs to AI. Yet also in Table 3, closely more than half (50.8%, 92/181) of this participants of this present study either think that AI does not pose any threat or are not sure if AI poses a threat to people's job security which in accordance with the results of [27] where a significant portion, 88.9% (*n* = 917/1032) of the respondents indicated that they were not afraid to lose their jobs due to AI implementation in radiology. This can be due to the fact that participants deeply understand the role of AI in radiology and it provides a positive outlook for AI adoption in medical imaging.

The study showed that most of the students have a fair knowledge of the application of AI in medical imaging. In Figure 1, almost three quarters (73.5%, 133/181) of second- to final-year students know that AI has been incorporated into current medical imaging modalities, and a majority (68.0%, 123/181) of them believe that AI helps to regulate radiation dose levels to patients while maintaining optimal image quality. This knowledge may be due to the fact that diagnostic imaging students undergo frequent clinical internships, helping them acquaint themselves with AI applications in imaging modalities. These findings are analogous to the findings of [26] where all the participants (*n* = 268/268) collectively indicated that there is the need for AI integration into various medical imaging modalities such as MRI, CT, PET, mammography, and general x-ray imaging.

Clinical internships in modern medical imaging should offer the student the opportunity to observe AI-powered modalities, such as those that help to detect anatomical landmarks to optimize patient positioning before exposure. Another common application is AI-based automatic positioning and centering in computed tomography, which is a new technique to reduce the dose and optimizing imaging workflow and image quality in chest imaging [28]. Therefore, it is surprising that (32.0%, 58/181) of the students believe that AI does not play a role in patient positioning from Figure 2. This ratio is quite significant considering this is the era of AI technology. This indicates why there is a need to continue to educate diagnostic imaging students in Ghana about AI and its prospects and applications in medical imaging. The finding is incidentally consistent with earlier work of [29] which shows that engineering students have poor knowledge about AI and its related fields.

### 5.3. Awareness of Artificial Intelligence in Medical Imaging

The study indicates that a significant percentage of diagnostic imaging students in Ghana are familiar with the concept of artificial intelligence (AI) in medical imaging. According to Table 4, 79.0% (143/181) of the students showed awareness of AI in medical imaging. This finding is consistent with similar research conducted in Turkey, which showed that most dental students had a general knowledge of AI in medical imaging [30] and similar to the findings of [22] where more than half (*n* = 138/263) of undergraduate medical students are aware that AI is a hot topic in radiology.

Most students (75.1%, 136/181) in Table 4 believe that AI will have an overall positive impact on medical imaging practice in Ghana. This result is consistent with the findings of [31] where (76.0%, 775/1020) practicing African radiographers were aware that the integration of AI into medical imaging practice in Africa would introduce more benefits than harm. This is a positive sign for the future of AI in healthcare in the country and Africa at large.

However, from Table 4, the research also revealed that almost half of the students (46.4%, 84/181) were unaware of the gradual emergence of AI in radiography in Ghana. This result is alarming and highlights the need for increased education among students to increase their level of awareness of AI applications in radiography in Ghana and beyond.

Interestingly, in Table 4, the studies showed that a significant number of diagnostic imaging students (64.7%, 117/181) believed that AI would not displace them from their jobs or are unsure if AI would someday replace them as radiographers. This result is consistent with the findings of a recent study by [20] that showed that 72.2% of practicing radiographers in Ghana disagreed or were neutral about the idea that AI would someday displace them from their job. This is a positive sign as it shows that students and professionals in the field of medical imaging in Ghana have a positive perception of the role of AI in healthcare. They understand that AI facilitates the work of radiographers and does not replace them.

Additionally, the current study from Table 4 shows that (64.6%, 117/181) of students agreed that there is a possibility of machine errors as a result of AI integration into medical imaging modalities. This assertion is similar to the findings of [31] which showed that (64.0%, 653/1020) African radiographers agreed that there is a possibility of errors associated with AI technologies integrated into clinical radiography. This indicates clearly that even though students are aware about the positive impact of AI in medical imaging, they show concern about the possibility of errors associated with AI technologies.

In conclusion, this study highlights the need to increase education and awareness about AI in radiography among diagnostic imaging students in Ghana. It also shows that the majority of students has a positive perception of the role of AI in medical imaging and do not see it as a threat to their job security. This is an encouraging sign for the future of AI in healthcare in Ghana.

### 5.4. Clinical Practice and Curriculum

With regards to AI education among diagnostic imaging students in Ghana, the findings is shown in Figure 2 of this study show that more than three-quarters (75.1%, 136/181) of the students believe that AI would improve education in medical imaging in Ghana. This is, however, lower than what has been reported in an earlier work of [20] among working radiographers where (94.7%, 143/151), of them believe that AI would improve radiography education here in Ghana. However, the current finding clearly indicates clearly that students know the potential benefits of artificial intelligence in medical imaging education. On the other side, 61.9% (112/181) of the students opinioned that by their experience in clinical internships in Ghana has not helped them to appreciate the role of AI in medical imaging, as shown in Figure 2. This assertion could be attributed partly to the fact that a number of health facilities may still be using older equipment with no AI technology incorporated. It may also be partly due to the fact that clinical instructors at those health facilities are not much aware of AI and require upgrading and training. All of these are indications that Ghana is still in the early stages of adopting AI technology in the medical imaging system.

The current undergraduate medical imaging curriculum lacks AI modules to help educate students about artificial intelligence. This fact was confirmed as more than three-quarters (76.2%, 138/181) of the students indicated that the current medical imaging teaching curriculum should incorporate AI modules from Figure 2. Similar recommendations have been made by doctors, radiologists, and medical and dental students who have suggested the need to include AI courses in the current medical education curriculum and residency training [1, 6, 23, 30, 32]. Additionally, a survey conducted in Canada among healthcare students by [33] showed that the majority of the respondents believed that gaining basic literacy in AI should be part of their curriculum. This was advocated to help in their medical training, and increase their level of knowledge and awareness about AI in medicine and healthcare. Fortunately, as shown in Figure 2, the medical imaging students showed enthusiasm towards AI. Nearly 90% (88.4%, 160/181) of the respondents said they were very interested in learning more about artificial intelligence and how it could be used in medical imaging.

This literature adds to the little existing literature on the knowledge and awareness of students about artificial intelligence. It is recommended that future studies include all medical imaging institutions in Ghana to help represent the true population of imaging students. Also, future studies should test for correlation or association between any demographic variable and the level of knowledge awareness among students. Lastly, it is recommended that the data collection period should be longer to ensure a larger sample size and more data to be gathered.

This study conducted in Ghana offers significant insights into the current state of knowledge and awareness of AI in medical imaging among diagnostic imaging students. It stands out as arguably the first of its kind conducted in the country, providing valuable information on the current state of understanding in this field. The study's findings hold great potential for guiding the development of educational interventions targeted at enhancing knowledge and awareness of AI in medical imaging. By utilizing these results, efforts can be directed toward designing effective educational programs that address any gaps and promote a deeper understanding of AI's role in the field of medical imaging.

It is important to consider certain limitations associated with this study. First, the sample size of medical imaging students was relatively small, which could potentially impact the generalizability of the findings. Additionally, the use of a self-administered and electronically administered questionnaire introduces the possibility of selection bias, which may have influenced the study's outcomes. Furthermore, it is essential to note that this study did not examine any correlation or association between demographic variables and the level of knowledge and awareness. Therefore, any potential relationships between these factors could not be explored or analyzed within the scope of this study. These limitations should be taken into account when interpreting the results and implications of this research.

## 6. Conclusions

This study offers valuable insights into the knowledge and perceptions of artificial intelligence (AI) in medical imaging. Participants showed awareness and understanding of AI's integration into imaging methods and its potential to enhance medical imaging. The study emphasizes the significance of addressing ethical and practical concerns such as displacement of radiographers and AI-machine errors, underlining the need for education, training, research, collaboration, and ethical implementation to harness AI's potential for revolutionizing healthcare.

## Figures and Tables

**Table 1:** Sample frame of participants.

School	Number of students
UCC	144
KNUST	159
UG	122
UHAS	91
Total (*n*)	516

**Table 2:** Sample frame of participants with respective sample sizes.

School	Number of year 2-year 4 students	Total number of year 2-year 4 students selected
UCC	144	63
KNUST	159	69
UG	122	53
UHAS	91	40
Total (*n*)	516	225

**Table 3:** Distribution of responses on general knowledge about artificial intelligence.

	Do you know the full meaning of AI (%)	Do you have knowledge about AI in general (%)	Do you think AI is a bad technology (%)	Do you think AI poses threat to people's job security (%)	Do you think AI is bringing about changes in the health sector (%)
Yes	92.3	72.9	3.3	49.2	81.2
No	6.6	17.1	71.8	20.4	5.5
Not sure	1.1	9.9	24.9	30.4	13.3

**Table 4:** Distribution of responses on awareness of artificial intelligence in medical imaging.

	Are you aware that AI is an emerging trend in medical imaging? (%)	Are you aware that AI is gradually emerging in Ghana's radiography sector (%)	Do you think AI would have an overall positive impact on medical imaging? (%)	Do you have the concern that AI would someday displace you from your work as a radiographer? (%)	Do you acknowledge the possibility of machine errors associated with AI-induced equipment? (%)
Yes	79.0	53.6	75.1	35.4	64.6
No	8.8	26.0	6.6	35.4	12.2
Not sure	12.2	20.4	18.2	29.3	23.2

## Data Availability

The datasets analyzed during the current study are available in Google Forms repository https://docs.google.com/forms/d/1ICZMB8B4cikecjucIzucMlE9e3ZAEfuTIJ_FCdzT5MI/edit#responses.

## Supplementary Materials

Supplementary Materials The accompanying supplementary file for this manuscript is a Questionnaire. The Questionnaire comprises the instrument used to collect data in the study, including the complete set of questions administered to participants.
