# Supplementary material for: Assessing the Level of Understanding (Knowledge) and Awareness of Diagnostic Imaging Students in Ghana on Artificial Intelligence and Its Applications in Medical Imaging
Source: Radiol Res Pract. 2023 Jun 15;2023:4704342. doi: 10.1155/2023/4704342 (PMC10287516; doi:10.1155/2023/4704342)
Supplement: Supplementary Materials — The accompanying supplementary file for this manuscript is a Questionnaire. The Questionnaire comprises the instrument used to collect data in the study, including the complete set of questions administered to participants. [file 4704342.f1.pdf]

APPENDIX 1 (RESEARCH INSTRUMENT)

SECTION A

PARTICIPANT INFORMATION SHEET

I am a level 400 Diagnostic Radiography student at the University of Cape Coast. I am carrying out a research study on the knowledge and awareness of diagnostic imaging students in Ghana on artificial intelligence and its prospects in medical imaging. The findings of this study will be used to provide baseline information for the various universities, and other relevant bodies about the level of knowledge and awareness diagnostic imaging students have about AI in medical imaging and it will also serve as a guide on how to educate the students. Participation in this study is voluntary and if at any point you wish to no longer take part in the research you have the right to withdraw at any time without any coercion. All the information you give **will be anonymous and confidential** and only used for the purposes of the research which will only be available only to me.

I would be most grateful if you could spare 15 minutes of your time to provide answers to the questionnaire.

Would you like to proceed with questionnaire?

- Agree
- Disagree

SECTION B

PART 1: PARTICIPANT'S DETAILS

Please provide the following information

Age: ....

Sex: ....

Level: ....

Name of institution: ....

PART 2: GENERAL KNOWLEDGE ABOUT ARTIFICIAL INTELLIGENCE.

In this part, kindly select either Yes or No

1. Do you know the full meaning of AI?

- Yes
- No
- Not sure

2. Do you have any knowledge about AI in general?

- Yes
- No
- Not sure

3. Do you think AI is a bad technology?

- Yes
- No
- Not sure

4. Do you think AI poses threat to people's job security?

- Yes
- No
- Not sure

5. Do you think AI is bringing about changes in the health sector?

- Yes
- No
- Not sure

PART 3: KNOWLEDGE ABOUT ARTIFICIAL INTELLIGENCE IN MEDICAL IMAGING

In this part, please provide either True or False.

6. AI has been incorporated into current imaging modalities such as CT machine, MRI, mammography, etc.

- True
- False
- Not sure

7. AI helps reduce radiation dose levels while maintaining optimal image quality in medical imaging.

- True
- False
- Not sure

8. AI plays a role in patient positioning and parameter selection during medical imaging procedures.

- True
- False
- Not sure

9. AI helps to detect pathologies in CT and MRI scans.

- True
- False
- Not sure

10. AI has increased the accuracy and sensitivity in the identification of chest pathologies.

- True
- False
- Not sure

PART 4: AWARENESS ABOUT ARTIFICIAL INTELLIGENCE IN MEDICAL IMAGING

<u>In this part, provide either Yes or No</u>

11. Are you aware that AI is an emerging trend in medical imaging?

- Yes
- No
- Not sure

12. Are you aware that AI is gradually emerging in Ghana's radiography sector?

- Yes
- No
- Not sure

13. Do you think AI would have an overall positive impact in medical imaging?

- Yes
- No
- Not sure

14. Do you have a concern that AI would someday displace you from your work as a radiographer?

- Yes
- No
- Not sure

15. Do you acknowledge the possibility of machine errors associated with AI- induced equipment in radiography unit.

- Yes
- No
- Not sure

PART 5: CLINICAL PRACTICE AND CURRICULUM

In this part, select either Yes or No.

16. Do you believe AI would improve education in medical imaging here in Ghana?

- Yes
- No
- Not sure

17. Do you think clinical practice helps you to appreciate the role of AI in medical imaging?

- Yes
- No
- Not sure

18. Do you think clinical practice helps to increase the level of knowledge and awareness about AI in medical imaging?

- Yes
- No
- Not sure

19. Do you think the current imaging curriculum in your school should incorporate modules on AI?

- Yes
- No
- Not sure

20. Would you like to learn more about AI and its prospects in medical imaging?

- Yes
- No
- Not sure
